# Latent profile and determinants of digital health literacy among older adult patients with chronic diseases: a cross-sectional study

**DOI:** 10.3389/fpubh.2025.1477314

**Published:** 2025-07-18

**Authors:** Yujiao Shao, Xuejun Xu, Hongyan Guo, Xiaocui Duan, Zeyu Zhang, Shuang Zhao, Xiumu Yang

**Affiliations:** ^1^School of Nursing, Bengbu Medical University, Bengbu, Anhui, China; ^2^Department of General Practice, First Affiliated Hospital of Bengbu Medical University, Bengbu, Anhui, China; ^3^Nursing Department, No. 902 Hospital of the People’s Liberation Army Joint Logistics and Security Force, Bengbu, Anhui, China; ^4^Nursing Department, First People's Hospital Affiliated with Bengbu Medical University, Bengbu, Anhui, China; ^5^General Practice Education Development Research Center, Bengbu Medical University, Bengbu, Anhui, China; ^6^School of Humanities and Social Sciences, University of Science and Technology of China, Hefei, Anhui, China

**Keywords:** older adult, chronic diseases, digital health literacy, influencing factors, latent profile analysis

## Abstract

**Objective:**

To examine the heterogeneity and determinants of digital health literacy among older adult patients with chronic diseases and provide evidence for targeted interventions.

**Methods:**

A convenience sample of 536 older adult patients with chronic diseases was recruited from three tertiary hospitals in Anhui Province between October 2023 and May 2024. Data were collected using structured questionnaires, including the Digital Health Literacy Assessment Scale, Social Support Rating Scale, General Self-Efficacy Scale, Brief Symptom Rating Scale, Brief Illness Perception Questionnaire, and the age-adjusted Charlson Comorbidity Index. Latent profile analysis (LPA) was conducted in Mplus 8.3. Univariate and multivariate logistic regression analyses were performed using SPSS 26.0 to identify literacy profiles and their associated factors.

**Results:**

The mean digital health literacy score was 41.36 (SD = 12.8), with an average item score of 2.76 (SD = 0.85). LPA identified three profiles: C1—Low Literacy, Passive Interaction (*n* = 142, 26.5%); C2—Moderate Literacy, Limited Interaction (*n* = 276, 51.5%); and C3—High Literacy, Active Interaction (*n* = 118, 22.0%). Multinomial logistic regression analysis showed that residence, participation in chronic disease health education, daily internet use, perceived ease of use and usefulness of digital health information, general self-efficacy, and social support were significant independent predictors of profile membership (*p* < 0.05). The model explained approximately 59.0% of the variance in profile classification (Nagelkerke *R*^2^ = 0.590).

**Conclusion:**

Digital health literacy among older adult patients with chronic diseases was generally low, particularly in interactive skills, with significant heterogeneity across subgroups. Tailored strategies that address the unique needs of each profile are essential to improve digital health literacy in this population.

## 1 Introduction

The global population is aging; by 2080, persons aged 65 or older will outnumber children under 18 (1). As the country with both the largest older adult population and one of the fastest aging rates globally, China faces substantial challenges linked to population aging. By the end of 2024, China’s population aged 60 and above had reached 310.31 million, representing 22% of the total population. Among them, 220.23 million were aged 65 or older, accounting for 15.6% (2). This share is projected to rise to 25% by 2050 (1), indicating that China has entered a moderately aging society (3). With age, cognitive, motor, and sensory functions decline, and mental health issues become more prominent. Over 78% of older adults have at least one chronic disease, and the number of disabled older adults is rising (3). Meanwhile, China’s healthcare system continues to struggle with the mismatch between limited high-quality medical resources and the increasingly complex health needs of its aging population (4). Older adult patients with chronic diseases require timely medical care, eldercare services, and reliable health information, yet traditional healthcare models no longer fully meet these demands.

As population aging accelerates, the incidence of chronic non-communicable diseases (NCDs) continues to grow. Characterized by high prevalence, long duration, low control rates, and high costs, chronic diseases have become a significant global public health burden. Each year, NCDs cause about 43 million deaths worldwide—75% of all deaths—including 18 million premature deaths (before age 70). Cardiovascular disease, cancer, chronic respiratory disease, and diabetes account for more than 80% of these deaths. In China, chronic diseases are highly prevalent among older adults, often accompanied by comorbidities and mental health problems, contributing to high rates of disability and mortality. Currently, cardiovascular and cerebrovascular diseases, cancer, chronic respiratory disease, and diabetes account for 88% of deaths in China and over 70% of the total disease burden. This poses a serious public health challenge with implications for national health and socioeconomic development (5). In response, improving chronic disease self-management has become a key strategy. Effective management slows disease progression, reduces complications, and improves quality of life. While demand for telemedicine, health management, and personalized medical services has surged in recent years, China still faces significant challenges in chronic disease prevention and control (3, 6).

In the context of accelerating digitalization, digital health governance is gaining global momentum (7), and China is rapidly advancing its digital health transition (4). As of December 2024, China had 1.108 billion internet users, with a penetration rate of 78.6%. Mobile users accounted for 1.105 billion, or 99.7% of total users. Among them, 61.2% demonstrated proficiency in at least one digital skill. Internet access among older adults reached 52.5%, with 47.4% capable of using “elder mode” features on mobile applications. Meanwhile, the internet healthcare sector expanded rapidly, with 418 million users—37.7% of total internet users—as service standardization and regulation continued to improve. Digital health technologies not only enhance health management efficiency but also drive the development of personalized care. The internet and social media have become key platforms for health information dissemination (8). Digital health literacy (DHL) refers to an individual’s ability to access, evaluate, interact with, and apply digital health information to maintain or improve health (9). Studies have shown that illness perception and social support significantly influence DHL (10, 11). In this study, illness perception was assessed using the Brief Illness Perception Questionnaire (BIPQ), which emphasizes the emotional and cognitive burden of disease. Therefore, higher BIPQ scores—indicating stronger perceived illness threat—may be associated with lower digital health literacy. Digital health interventions can improve self-management in patients with chronic diseases (12), thereby enhancing health outcomes and quality of life (13). According to social ecological systems theory (14, 15), individual development is shaped by multilayered systems, including the microsystem (individual factors such as self-efficacy, illness perception, internet use habits), the mesosystem (interpersonal factors such as participation in health education and perceived social support), and the macrosystem (societal and technological contexts such as digital infrastructure and the usability and usefulness of digital health platforms). This framework enables a comprehensive understanding of how personal, social, and contextual factors interact to influence digital health literacy in older adults with chronic diseases.

Most current domestic studies on DHL adopt a variable-centered approach, assuming population homogeneity and ignoring individual-level differences. This limits the identification of unique characteristics and needs. However, developing effective DHL interventions requires recognizing the heterogeneity of the target population. Latent Profile Analysis (LPA), a person-centered method, identifies shared patterns of responses, classifies individuals into subgroups, and reveals underlying population heterogeneity, thereby improving the precision of classification (16, 17). This study applies LPA to explore DHL profiles among older adult patients with chronic diseases and, based on social ecological systems theory, examines the influencing factors associated with each profile to inform tailored intervention strategies.

## 2 Participants and methods

### 2.1 Participants

This study used a convenience sampling method to recruit older adult patients with chronic diseases from three tertiary hospitals in Anhui Province between October 2023 and May 2024. Inclusion criteria were as follows: (1) age ≥60 years; (2) diagnosis of a chronic disease according to the ICD-10, including but not limited to hypertension, diabetes, cancer, chronic respiratory diseases, and coronary artery disease; (3) ability to use smart devices (e.g., access the internet via mobile phone); (4) normal consciousness and the ability to read or communicate; and (5) voluntary participation with informed consent. Exclusion criteria included: (1) severe cognitive, speech, visual, auditory, or psychiatric impairments; (2) acute or critical illness with serious complications or organ failure during the study; (3) inability to engage in extended communication or having significant communication barriers; and (4) simultaneous participation in other related studies.

Sample size was estimated using the standard formula for cross-sectional studies: N (*μ*_α/2_*σ*/*δ*)^2^ (18), where α = 0.05, *μ*_α/2_ = 1.96, *δ* = allowable error, and *σ* = standard deviation. A pilot survey (*n* = 40) reported mean scores and standard deviations for digital health literacy (2.76 ± 0.85), social support (2.97 ± 0.63), general self-efficacy (2.26 ± 0.60), psychological status (2.16 ± 0.72), and illness perception (5.93 ± 0.88). The maximum standard deviation (*σ* = 0.88) was selected. With *δ* set to 0.25σ (19) (i.e., 0.22), the calculated minimum sample size was 62. Accounting for a 20% attrition rate, the adjusted sample size was 75. According to multivariable analysis guidelines (20), the recommended sample size is 5–10 times the number of variables. With 30 variables and a 20% attrition buffer, the target sample size was 180–360. Moreover, Nylund-Gibson et al. (21) emphasize that latent profile analysis (LPA) requires a sample size of at least 300 to ensure convergence and capture small profile groups. In total, 536 participants were included, satisfying the requirements for LPA. Ethical approval was obtained from the Ethics Committee of Bengbu Medical University (Approval No. [2023]369).

### 2.2 Research tools

#### 2.2.1 General information questionnaire

This questionnaire was developed by the research team through literature review and consultation with clinical experts. It collected data on demographics (e.g., age, gender, education), disease characteristics (e.g., number of chronic conditions, self-rated health), and internet use, including digital device usage and attitudes toward digital health information.

#### 2.2.2 Digital health literacy assessment scale (DHLS)

Developed by Liu (9), the DHLS consists of 15 items across three dimensions: the ability to access and evaluate digital health information, interaction ability, and application ability. Total scores range from 15 to 75, with higher scores indicating higher digital health literacy. The scale’s Cronbach’s *α* was 0.941 and 0.906 in the present study.

#### 2.2.3 Social support rating scale (SSRS)

Developed by Xiao (22), the SSRS includes 10 items covering three dimensions: objective support, subjective support, and support utilization. Total scores range from 12 to 83, with higher scores reflecting greater social support. Cronbach’s *α* was 0.896 in previous studies (23), and 0.720 in this study.

#### 2.2.4 10-item Kessler psychological distress scale (K10)

The K10, developed by Kessler (24) and translated into Chinese by Zhou et al. (25), assesses psychological distress over the past month. It includes 10 items, with total scores ranging from 10 to 50; higher scores indicate greater distress. Cronbach’s *α* was 0.8011 (26) and 0.900 in this study.

#### 2.2.5 General self-efficacy scale (GSES)

Developed by Schwarzer et al. (27) and translated by Wang et al. (28), the 10-item GSES measures confidence in handling challenges. Total scores range from 10 to 40, with higher scores indicating stronger perceived self-efficacy. Cronbach’s *α* was 0.871 in previous studies and 0.873 in this study.

#### 2.2.6 Brief illness perception questionnaire (BIPQ)

The BIPQ, developed by Broadbent et al. (29) and translated by Sun et al. (30), includes nine items measuring cognitive and emotional illness representations, illness understanding, and perceived causes. This study included only the first three dimensions, excluding the open-ended question on perceived causes. Total scores range from 0 to 80, with higher scores reflecting more negative illness perceptions. Cronbach’s *α* was 0.831 in prior studies and 0.751 here.

#### 2.2.7 Age-adjusted Charlson comorbidity index (aCCI)

The aCCI quantifies comorbidity severity by incorporating both age and the number of chronic conditions. Disease scores are assigned values of 1, 2, 3, or 6 based on specific conditions. Age categories (<50, 50–59, 60–69, 70–79, 80–89, ≥90 years) are scored as 0–5, respectively. Higher scores indicate greater comorbidity burden (31, 32). Disease data were verified via electronic medical records and physician evaluations.

### 2.3 Data collection

Data were collected from the outpatient and inpatient departments of three tertiary hospitals in Anhui Province, China. The sample included both inpatients and patients attending regular follow-up visits for chronic disease management. Prior to recruitment, the research team obtained formal approval from the relevant hospital departments.

To enhance cultural and contextual sensitivity, the investigators—who were also trained clinical nurses—participated in routine care activities, enabling them to build rapport with patients and become familiar with their language habits and cultural backgrounds. Eligible participants were approached in a private setting, where the study’s purpose, procedures, confidentiality measures, and audio recording requirements were clearly explained in plain and accessible language. Written informed consent was obtained from all participants before enrollment.

After obtaining consent, participants completed the questionnaires independently in a quiet setting. For those with visual, literacy, or physical difficulties, trained researchers provided one-on-one assistance to ensure understanding without influencing responses. Additionally, disease-related information was verified and supplemented through medical record review.

A total of 550 eligible older adult patients were invited to participate, of whom 536 completed the questionnaire, yielding a response rate of 97.45%. The dropout rate was 2.25%, primarily due to invalid responses—such as patterned answering (e.g., identical scores across items), extreme values, or multiple selections in single-choice items. Completed questionnaires were collected immediately upon completion and reviewed on-site for completeness. Any missing or ambiguous responses were clarified in real time with the participants. Questionnaires were deemed invalid if they contained over 10% patterned or extreme responses or multiple answers to single-choice questions, as defined in previous literature (33, 34).

To minimize expectation bias, participation was entirely voluntary, and respondents were informed that their answers would remain anonymous and have no impact on their care. Researchers provided assistance only in terms of questionnaire delivery or completion support (e.g., reading questions), without influencing the content of responses.

### 2.4 Statistical methods

Data were double-checked, organized, and entered into a database. Latent profile modeling was conducted in Mplus 8.3 using the 15 DHLS items as observed variables. Models with increasing class numbers were tested, and the optimal solution was selected based on model fit and clinical interpretability. Evaluation criteria included:

① Information criteria—AIC, BIC, and adjusted BIC (aBIC), with lower values indicating better fit; ② Classification accuracy—Entropy values (range: 0–1), with values closer to 1 indicating better precision; ③ Likelihood ratio tests—Lo–Mendell–Rubin (LMR) and bootstrap likelihood ratio test (BLRT), with *p* < 0.05 indicating that the K-class model outperforms the K − 1 class model. Common method bias was assessed using Harman’s single-factor test in SPSS 26.0. Skewness and kurtosis tested univariate normality; Mardia’s test assessed multivariate normality. Variables with normal or near-normal distributions were reported as mean ± SD and compared using one-way ANOVA. Categorical variables were expressed as frequencies or percentages and analyzed using *χ*^2^ or Fisher’s exact test. Variables significant in univariate analysis were included in multivariate logistic regression. A *p*-value < 0.05 was considered statistically significant.

## 3 Results

### 3.1 Common method bias test

Given the self-reported nature of the data, there was potential for common method bias. To mitigate this, participants were assured of anonymity and confidentiality before the survey. During the survey, item order was balanced, and disease-related information was corroborated through multiple sources, including electronic medical records, to reduce single-source bias. Harman’s single-factor test was employed, revealing 13 factors with eigenvalues exceeding 1. The first factor accounted for 22.049% of the variance, below the 40% threshold (35), indicating that common method bias was not a significant concern.

### 3.2 Normality test

Univariate normality was evaluated using skewness and kurtosis. The absolute values of skewness for all variables were less than 3, and kurtosis values were below 10, meeting Kline’s criteria and suggesting an approximately normal distribution (36). Multivariate normality was assessed using the Mardia test. Additionally, the standardized multivariate kurtosis coefficient (|std-MK|) was 2.4423, which satisfies Byrne’s criterion of std-MK < 5 for multivariate normality (37) (see Table 1).

**Table 1:** Statistics of normality.

Variables	Skewness	Skewness SE	Kurtosis	Kurtosis SE
DHL	−0.021	0.106	−0.752	0.211
SSRS	0.338	0.106	−0.184	0.211
GSES	−0.015	0.106	−1.045	0.211
K10	1.039	0.106	1.486	0.211
BIPQ	−0.071	0.106	0.008	0.211
aCCI	0.625	0.106	−0.141	0.211

### 3.3 General information of the participants

**Figure 1:**
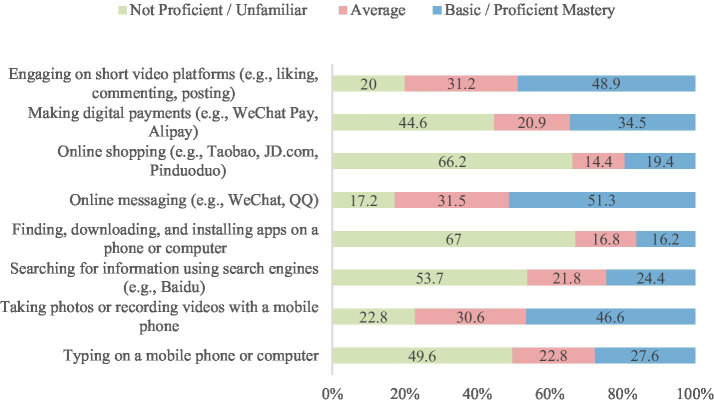
Proficiency levels in selected digital literacy skills among participants.

A total of 550 questionnaires were distributed; 14 were invalid, resulting in 536 valid responses and an effective response rate of 97.45%. The participants’ mean age was 67.4 ± 6.9 years. Additional demographic details are presented in Table 2. As shown in Table 3, hypertension was the most prevalent chronic disease among participants. The differences in chronic disease types were statistically significant (*χ*^2^ = 1,010.71, df = 11, *p* < 0.0001). Table 4 indicates that smartphones were the most commonly used smart devices, with usage differences also statistically significant (*χ*^2^ = 1,436.67, df = 7, *p* < 0.0001). Regarding digital literacy, Figure 1 illustrates that the highest proficiency rates were in online chatting (51.30%), interacting on short video platforms (48.90%), and taking photos/videos with a mobile phone (46.60%). Lower proficiency was observed in online shopping (19.40%) and software downloading/installation (16.20%). This suggests a stratification in digital skills, with social and entertainment-related abilities being more developed than utilitarian and complex operational skills.

**Table 2:** Univariate analysis of general characteristics and latent categories of digital health literacy among older adult patients with chronic diseases.

Variables		Number (%)	C1	C2	C3	*χ*^2^/*F*	*p*
			(*n* = 142)	(*n* = 276)	(*n* = 118)		
Age	60–64	238 (44.4)	46 (32.4)	118 (42.8)	74 (62.7)	31.846	<0.001
	65–69	101 (18.8)	24 (16.9)	57 (20.7)	20 (16.9)		
	70–74	107 (20.0)	39 (27.5)	53 (19.2)	15 (12.7)		
	75–79	59 (11.0)	22 (15.5)	30 (10.9)	7 (5.9)		
	≥80	31 (5.8)	11 (7.7)	18 (6.5)	2 (1.7)		
Gender	Male	304 (56.7)	83 (58.5)	152 (55.1)	69 (58.5)	0.626	0.731
	Female	232 (43.3)	59 (41.5)	124 (44.9)	49 (41.5)		
Place of residence	Rural	86 (16.0)	40 (28.2)	42 (15.2)	4 (3.4)	65.636	<0.001
	Town	128 (23.9)	53 (37.3)	57 (20.7)	18 (15.3)		
	City	322 (60.1)	49 (34.5)	177 (64.1)	96 (81.4)		
Education level	Primary school or below	207 (38.6)	94 (66.2)	107 (38.8)	6 (5.1)	191.012	<0.001
	Middle school	178 (33.2)	39 (27.5)	107 (38.8)	32 (27.1)		
	High/Vocational school	97 (18.1)	9 (6.3)	50 (18.1)	38 (32.2)		
	College or above	54 (10.1)	0 (0)	12 (4.3)	42 (35.6)		
Marital status	Married	445 (83.0)	112 (78.9)	222 (80.4)	111 (94.1)	13.259	0.001
	Divorced/Widowed/Other	91 (17.0)	30 (21.1)	54 (19.6)	7 (5.9)		
Multiple-child	No	233 (43.5)	23 (16.2)	123 (44.6)	87 (73.7)	87.082	<0.001
	Yes	303 (56.5)	119 (83.8)	153 (55.4)	31 (26.3)		
Living situation	Spouse	318 (59.3)	79 (55.6)	166 (60.1)	73 (61.9)	13.493	0.036
	(Grandson) children	44 (8.2)	19 (13.4)	20 (7.2)	5 (4.2)		
	Spouses and (grandchildren) children	99 (18.5)	27 (19)	44 (15.9)	28 (23.7)		
	Alone	75 (14)	17 (12)	46 (16.7)	12 (10.2)		
Occupation before retirement	Farmer	162 (30.2)	87 (61.3)	72 (26.1)	3 (2.5)	165.398^#^	<0.001
	Worker	74 (13.8)	14 (9.9)	55 (19.9)	5 (4.2)		
	State-owned enterprise/Institution/Civil servant	167 (31.2)	13 (9.2)	81 (29.3)	73 (61.9)		
	Private enterprise employee	52 (9.7)	11 (7.7)	27 (9.8)	14 (11.9)		
	Private enterprise employee	71 (13.2)	16 (11.3)	35 (12.7)	20 (16.9)		
	Other	10 (1.9)	1 (0.7)	6 (2.2)	3 (2.5)		
Medical insurance type	Employee insurance	292 (54.5)	43 (30.3)	160 (58)	89 (75.4)	77.637^#^	<0.001
	Resident insurance	231 (43.1)	98 (69)	112 (40.6)	21 (17.8)		
	Commercial insurance or other	13 (2.4)	1 (0.7)	4 (1.4)	8 (6.8)		
Average monthly income (RMB)	<1,000	130 (24.3)	75 (52.8)	50 (18.1)	5 (4.2)	143.956	<0.001
	1,000–2,999	101 (18.8)	27 (19)	60 (21.7)	14 (11.9)		
	3,000–4,999	135 (25.2)	20 (14.1)	91 (33)	24 (20.3)		
	≥5,000	170 (31.7)	20 (14.1)	75 (27.2)	75 (63.6)		
Number of chronic diseases	One	148 (27.6)	31 (21.8)	72 (26.1)	45 (38.1)	9.233	0.010
	Two or more	388 (72.4)	111 (78.2)	204 (73.9)	73 (61.9)		
Duration of illness *(Years)	≤1	85 (15.9)	25 (17.6)	40 (14.5)	20 (16.9)	4.832	0.775
	(1–3]	104 (19.4)	30 (21.1)	47 (17)	27 (22.9)		
	(3–5]	59 (11.0)	14 (9.9)	30 (10.9)	15 (12.7)		
	(5–10]	62 (11.6)	15 (10.6)	34 (12.3)	13 (11)		
	>10	226 (42.2)	58 (40.8)	125 (45.3)	43 (36.4)		
Self-rated health status	Good/very good	243 (45.3)	51 (35.9)	114 (41.3)	78 (66.1)	37.178	<0.001
	Average	196 (36.6)	50 (35.2)	114 (41.3)	32 (27.1)		
	Bad/very bad	97 (18.1)	41 (28.9)	48 (17.4)	8 (6.8)		
Self-rated disease control status	Good/very good	255 (47.6)	49 (34.5)	126 (45.7)	80 (67.8)	34.094	<0.001
	Average	179 (33.4)	53 (37.3)	96 (34.8)	30 (25.4)		
	Bad/very bad	102 (19)	40 (28.2)	54 (19.6)	8 (6.8)		
Attended chronic disease health lectures	Yes	290 (54.1)	64 (45.1)	148 (53.6)	78 (66.1)	11.533	0.003
	No	246 (45.9)	78 (54.9)	128 (46.4)	40 (33.9)		
Number of digital devices used	One	138 (25.7)	56 (39.4)	73 (26.4)	9 (7.6)	34.257	<0.001
	Two or more	398 (74.3)	86 (60.6)	203 (73.6)	109 (92.4)		
Average daily online time (h)	≤1	137 (25.6)	77 (54.2)	55 (19.9)	5 (4.2)	143.496	<0.001
	(1–2]	154 (28.7)	32 (22.5)	102 (37)	20 (16.9)		
	(2–3]	104 (19.4)	16 (11.3)	59 (21.4)	29 (24.6)		
	(3–4]	85 (15.9)	7 (4.9)	42 (15.2)	36 (30.5)		
	>4	56 (10.4)	10 (7)	18 (6.5)	28 (23.7)		
Perception of digital health information							
Usefulness	Not useful/Somewhat useful	101 (18.8)	52 (36.6)	40 (14.5)	9 (7.6)	44.071	<0.001
	Average	157 (29.3)	27 (19)	88 (31.9)	42 (35.6)		
	Quite/Very useful	278 (51.9)	63 (44.4)	148 (53.6)	67 (56.8)		
Ease of use	Very/Quite difficult	207 (38.6)	105 (73.9)	96 (34.8)	6 (5.1)	203.426	<0.001
	Average	183 (34.1)	28 (19.7)	124 (44.9)	31 (26.3)		
	Quite/Very easy	146 (27.2)	9 (6.3)	56 (20.3)	81 (68.6)		
Risk degree	Very/relatively small	233 (43.5)	75 (52.8)	102 (37)	56 (47.5)	30.057	<0.001
	Average	157 (29.3)	20 (14.1)	93 (33.7)	44 (37.3)		
	Compare/very high	146 (27.2)	47 (33.1)	81 (29.3)	18 (15.3)		
Trust degree	Very/not too sure	108 (20.1)	46 (32.4)	54 (19.6)	8 (6.8)	30.042	<0.001
	Average	171 (31.9)	31 (21.8)	90 (32.6)	50 (42.4)		
	Compare/strongly believe	257 (47.9)	65 (45.8)	132 (47.8)	60 (50.8)		
Attitudes toward help-seeking in internet use							
Ask for help	Never/rarely	279 (52.1)	89 (62.7)	113 (40.9)	77 (65.3)	29.122	<0.001
	Sometimes	226 (42.2)	46 (32.4)	142 (51.4)	38 (32.2)		
	Often/always	31 (5.8)	7 (4.9)	21 (7.6)	3 (2.5)		
Passive dependence	Never/rarely	255 (47.6)	70 (49.3)	110 (39.9)	75 (63.6)	19.704	0.001
	Sometimes	232 (43.3)	57 (40.1)	138 (50)	37 (31.4)		
	Often/always	49 (9.1)	15 (10.6)	28 (10.1)	6 (5.1)		

*For patients with multiple chronic diseases, the duration is based on the first diagnosis.

**Table 3:** Frequency of reported chronic diseases (*n* = 536).

Chronic disease	Number of responses	Response percentage (%)	Percentage of cases (%)
Hypertension	357	29.50	66.60
Diabetes	171	14.10	31.90
Hyperlipidemia	26	2.10	4.90
Chronic liver disease	35	2.90	6.50
Chronic kidney disease	28	2.30	5.20
Cancer	82	6.80	15.30
Chronic respiratory diseases	65	5.40	12.10
Cardiovascular and cerebrovascular diseases	180	14.90	33.60
Bone and joint diseases	85	7.00	15.90
Neurological diseases	20	1.70	3.70
Gastrointestinal diseases	63	5.20	11.80
Others	98	8.10	18.30
Total	1,210	100.00	225.70

**Table 4:** Frequency of digital device usage (*n* = 536).

Digital device used	Number of responses	Response percentage (%)	Percentage of cases (%)
Smartphones	533	42.60	99.40
Smart speakers	17	1.40	3.20
Digital televisions	220	17.60	41.00
Computers/tablets	48	3.80	9.00
Smart wearable devices	60	4.80	11.20
Electronic blood pressure monitors	269	21.50	50.20
Glucometers	93	7.40	17.40
Others	10	0.80	1.90
Total	1,250	100.00	233.20

### 3.4 Latent profile analysis and profile naming

**Figure 2:**
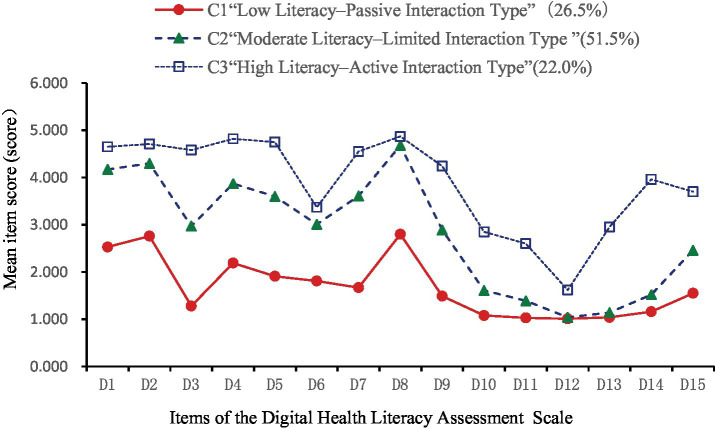
Latent profile chart of digital health literacy among older adult patients with chronic diseases.

Utilizing the 15 items of the Digital Health Literacy Scale as indicators, latent profile analysis (LPA) was conducted with Mplus 8.3. Models with 1 to 5 classes were evaluated (see Table 5). As the number of classes increased, AIC, BIC, and aBIC values decreased, indicating improved fit. Entropy values exceeded 0.9, suggesting high classification accuracy. The LMR test for the 4-class model was not statistically significant (*p* > 0.05), indicating no superiority over the 3-class model. In the 3-class model, entropy was 0.941, with significant LMR and BLRT tests (*p* < 0.05). Class sizes were 142, 276, and 118, each exceeding 5% of the sample. Considering fit indices, sample distribution, and clinical relevance, the 3-class model was selected. Table 6 presents the mean scores of digital health literacy for the three profiles. Figure 2 depicts the profile plot, leading to the following classifications:

**Table 5:** Latent profile analysis models and fit indices.

Model	K	AIC	BIC	aBIC	Entropy	LMR (*p*)	BLRT (*p*)	Proportion
1	30	26,133.729	26,262.253	26,167.023	–	–	–	1.000
2	46	23,659.931	23,857.001	23,710.982	0.917	0.0001	<0.001	0.511/0.489
3	62	22,559.956	22,825.572	22,628.764	0.941	0.0001	<0.001	0.265/0.515/0.220
4	78	21,902.264	22,236.426	21,988.828	0.934	0.3100	<0.001	0.213/0.396/0.313/0.078
5	94	21,429.781	21,832.489	21,534.102	0.939	0.0869	<0.001	0.194/0.370/0.110/0.277/0.049

K represents the number of freely estimated parameters.

**Table 6:** Digital health literacy and scores of each dimension of the three potential profiles.

Variables	Number of items		Score	C1	C2	C3
				(*n* = 142)	(*n* = 276)	(*n* = 118)
Digital health literacy scale	15	a	41.36 ± 12.8	25.3 ± 5.45	42.39 ± 5.95	58.29 ± 5.05
		b	2.76 ± 0.85	1.69 ± 0.36	2.83 ± 0.4	3.89 ± 0.34
Access and evaluation of digital health information	9	a	30.9 ± 9.27	18.45 ± 4.76	33.19 ± 5.02	40.53 ± 3.53
		b	3.43 ± 1.03	2.05 ± 0.53	3.69 ± 0.56	4.5 ± 0.39
Interaction ability	3	a	4.48 ± 2.14	3.13 ± 0.47	4.05 ± 1.38	7.08 ± 2.57
		b	1.49 ± 0.71	1.04 ± 0.16	1.35 ± 0.46	2.36 ± 0.86
Application ability	3	a	5.99 ± 3.27	3.73 ± 1.7	5.14 ± 1.93	10.68 ± 2.58
		b	2 ± 1.09	1.24 ± 0.57	1.72 ± 0.64	3.56 ± 0.86

a = total score, b = item mean score.

C1: Low Literacy–Passive Interaction Type (142 participants, 26.5%): Consistently low scores across all items, notably in information acquisition and evaluation. Item 3 (“Actively searching for needed health information online”) was particularly low, indicating poor proactive information-seeking behavior. Despite low overall scores, item 8 was relatively higher, suggesting that participants considered the relevance of information to their conditions. Interaction abilities (items 10–12) were weak, especially in online sharing and communication. Application skills (items 13–15) were also low, notably in using online consultation functions. This group exhibited low digital health literacy with a tendency for passive information reception. C2: Moderate Literacy–Limited Interaction Type (276 participants, 51.5%): Moderate scores across all items, with improvements in information acquisition and evaluation (items 1–9). However, interaction abilities (items 10–12) remained relatively low, indicating limited engagement in online information exchange. Application skills showed enhancements, particularly in monitoring health indicators and accessing medical services digitally. This group demonstrated moderate digital health literacy with restricted interaction capabilities. C3: High Literacy–Active Interaction Type (118 participants, 22.0%): High scores across all items, especially in proactive health information acquisition (item 3). While item 12 scores suggested cautiousness in activities like online voting, overall interaction abilities were strong. Application skills were well-developed, indicating a high level of digital health literacy with active engagement in digital health activities.

### 3.5 Univariate analysis of the latent profiles of digital health literacy in older adult patients with chronic diseases

The univariate analysis revealed significant differences (*p* < 0.05) among the latent profiles in variables including age, place of residence, education level, marital status, number of children, cohabitation status, pre-retirement occupation, type of medical insurance, per capita monthly household income, number of chronic conditions, self-rated health status, self-rated disease control, participation in chronic disease health education, number of smart devices used, average daily internet use, perception of digital health information, and attitudes toward online health-seeking behavior (Table 2). Significant differences (*p* < 0.05) were also observed in total and subscale scores for social support, general self-efficacy, and illness perception (including cognitive representation and illness understanding) across the three latent profiles (Table 7).

**Table 7:** Comparison of scores of variables in three profiles.

Variables	Score	C1	C2	C3	Multiple comparisons	*F*	*p*
		*n* = 142	*n* = 276	*n* = 118			
SSRS	29.67 ± 6.3	27.08 ± 5.13	29.26 ± 5.91	33.73 ± 6.52	C1 < C2 < C3	42.811	<0.001
Objective support	8.91 ± 2.46	8.39 ± 2.28	8.57 ± 2.27	10.34 ± 2.6	C1 < C3, C2 > C3	28.25	<0.001
Subjective support	14.13 ± 3.66	12.58 ± 2.72	14.05 ± 3.55	16.16 ± 3.95	C1 < C2 < C3	34.792	<0.001
Support utilization	6.63 ± 1.95	6.11 ± 1.88	6.64 ± 1.92	7.23 ± 1.96		10.922	<0.001
GSES	2.27 ± 0.61	1.93 ± 0.54	2.27 ± 0.59	2.66 ± 0.46	C1 < C2 < C3	56.387	<0.001
K10	23.87 ± 7.76	21.55 ± 6.68	18.93 ± 6.6	23.87 ± 7.76	C3 < C2 < C1	16.178	<0.001
BIPQ	47.4 ± 7.05	49.36 ± 6.78	47.41 ± 6.67	45.03 ± 7.54	C1 > C2 > C3	12.667	0.001
Cognitive and emotional illness representations	28.97 ± 5.19	30.75 ± 4.84	28.8 ± 5.08	27.2 ± 5.22		16.137	0.002
Illness understanding	13.04 ± 2.11	13.01 ± 2.1	13.22 ± 2.04	12.66 ± 2.21	C2 > C3	2.971	0.052
Perceived causes	5.4 ± 1.3	5.61 ± 1.37	5.38 ± 1.26	5.17 ± 1.28	C1 > C3	3.669	0.026
aCCI	3.4 ± 1.23	3.8 ± 1.36	3.43 ± 1.13	2.85 ± 1.08	C1 > C2 > C3	20.987	<0.001

### 3.6 Multivariate analysis of the latent profiles of digital health literacy in older adult patients with chronic diseases

Multinomial logistic regression was conducted using the latent profiles of digital health literacy (C1 = 1, C2 = 2, C3 = 3) as the dependent variable. Independent variables included all factors found significant in the univariate analysis. Original scores were used for social support, general self-efficacy, psychological distress, illness perception, and aCCI. Coding for other variables is detailed in Table 8. Results (Table 9) showed that, compared to C1 and C2, participants who had attended health education sessions and had higher levels of self-efficacy and social support were more likely to be classified as C3. Compared to C3, individuals living in urban areas, using the internet less than 1 h daily, perceiving digital health information as “not at all/slightly useful,” reporting usability as “very/somewhat difficult” or “neutral,” and having higher aCCI scores were more likely to belong to C1. Similarly, those with daily internet use up to 3 h, who rated digital health information as “not at all/slightly useful,” and perceived usability as “very/somewhat difficult” or “neutral,” with higher aCCI scores, were more likely to be in C2. Compared to C2, participants living in rural or urban areas, perceiving digital health information as “not at all/slightly useful,” and rating usability as “very/somewhat difficult,” were more likely to belong to C1. In contrast, attending health education sessions, using the internet for 1–4 h daily, and having higher self-efficacy scores increased the likelihood of being in C2.

**Table 8:** Assignment of independent variables in multiple logistic regression.

Variables	Assignment of variables
Age	60–64 = 1, 65–69 = 2, 70–74 = 3, 75–79 = 4, ≥80 = 5
Education level	Primary school or below = 1, Middle school = 2, High/Vocational school = 3, College or above = 4
Place of residence	Rural = 1, Town = 2, City = 3
Attended chronic disease health lectures	Yes = 1, No = 2
Number of digital devices used	one = 1, Two or more = 2
Average daily online time (h)	≤1 = 1, (1–2] = 2, (2–3] = 3, (3–4] = 4, >4 = 5
Perception of digital health information	
Usefulness	Not useful/Somewhat useful = 1, Average = 2, Quite/Very useful = 3
Ease of Use	Very/Quite difficult = 1, Average = 2, Quite/Very easy = 3
Risk degree	Very/relatively small = 1, Average = 2, Compare/very high = 3
Trust degree	Very/not too sure = 1, Average = 2, Compare/strongly believe = 3
Attitudes toward help-seeking in internet use	
Ask for help	Never/rarely = 1, Sometimes = 2, Often/always = 3
Passive dependence	Never/rarely = 1, Sometimes = 2, Often/always = 3

**Table 9:** Multiple logistic regression analysis of latent categories.

	*β*	SE	waldχ2	*p*	OR	95%CI
C1 vs C3*						
Intercept	3.155	1.396	5.108	0.024		
Town	1.001	0.451	4.921	0.027	2.721	1.124–6.59
Attended chronic disease health lectures	−1.246	0.375	11.026	0.001	0.288	0.138–0.6
Average daily online time (<1 h)	2.468	0.744	10.996	0.001	11.795	2.743–50.715
Perception of digital health information						
Usefulness (not useful/Somewhat useful)	2.196	0.569	14.878	0.000	8.985	2.944–27.419
Ease of use (very/quite difficult)	3.96	0.631	39.351	0.000	52.454	15.221–180.762
Ease of use (average)	1.797	0.514	12.237	0.000	6.031	2.204–16.504
GSES	−1.377	0.355	15.075	0.000	0.252	0.126–0.506
SSRS	−0.113	0.033	11.785	0.001	0.893	0.837–0.953
aCCI	0.407	0.161	6.413	0.011	1.502	1.096–2.057
C2 vs C3*						
Intercept	2.646	1.086	5.932	0.015		
Attended chronic disease health lectures	−0.629	0.298	4.446	0.035	0.533	0.297–0.957
Average daily online time (<1 h)	2.334	0.629	13.788	0.000	10.324	3.011–35.397
Average daily online time [(1–2] hours]	1.637	0.47	12.133	0.000	5.139	2.046–12.909
Average daily online time [(2–3] hours]	1.079	0.46	5.498	0.019	2.941	1.194–7.248
Perception of digital health information						
Usefulness (not useful/Somewhat useful)	1.031	0.503	4.19	0.041	2.803	1.045–7.518
Ease of use (very/quite difficult)	2.547	0.501	25.811	0.000	12.765	4.779–34.098
Ease of use (average)	1.443	0.314	21.085	0.000	4.232	2.286–7.835
GSES	−0.887	0.293	9.155	0.002	0.412	0.232–0.732
SSRS	−0.075	0.024	9.855	0.002	0.928	0.885–0.972
aCCI	0.32	0.131	5.999	0.014	1.378	1.066–1.78
C2 vs C1*						
Intercept	−0.509	1.028	0.246	0.620		
Rural	−0.789	0.329	5.75	0.016	0.454	0.238–0.866
Town	−0.968	0.299	10.454	0.001	0.38	0.211–0.683
Attended chronic disease health lectures	0.617	0.258	5.734	0.017	1.853	1.119–3.071
Average daily online time [(1–2] hours]	1.247	0.52	5.749	0.016	3.478	1.256–9.635
Average daily online time [(3–4] hours]	1.457	0.626	5.41	0.020	4.291	1.258–14.644
Perception of digital health information						
Usefulness (not useful/Somewhat useful)	−1.165	0.318	13.46	0.000	0.312	0.167–0.581
Ease of use (very/quite difficult)	−1.413	0.432	10.688	0.001	0.243	0.104–0.568
GSES	0.49	0.23	4.543	0.033	1.633	1.04–2.564

*For the reference group; The highest assignment was used as the control for all independent variables.

Multinomial logistic regression analysis showed that residence, participation in chronic disease health education, daily internet use, perceived ease of use and usefulness of digital health information, general self-efficacy, and social support were significant independent predictors of profile membership (*p* < 0.05). The model explained approximately 59.0% of the variance in profile classification (Nagelkerke *R*^2^ = 0.590).

## 4 Discussion

### 4.1 Status of digital health literacy in older adult patients with chronic conditions

#### 4.1.1 Overall level of digital health literacy

This study found that the mean digital health literacy score among older adult patients with chronic diseases was 41.36 ± 12.8, with an average item score of 2.76 ± 0.85 (range: 1–5). This score falls below the standard median of 3, suggesting generally low digital health literacy. However, it is slightly higher than those reported in previous studies of older adults in rural or community-based settings in China. Yang et al. (38) reported a score of 39.76 ± 13.82 (average item score: 2.65 ± 0.92) among rural older adults in Hebei Province. A study in rural Chengde (39) found a score of 34.90 ± 17.18 (item average: 2.33 ± 1.15). Liu (9) reported a total score of 37.10 ± 18.65 (item average: 2.47 ± 1.68) among community-dwelling older adults in Chongqing. These previous results suggest slightly lower digital health literacy levels compared to our findings. The discrepancy may stem from differences in sample characteristics. In our study, only 16.0% of participants were from rural areas, suggesting that rural older adults may have lower digital health literacy than their urban counterparts. Moreover, all participants were hospital outpatients, likely leading to greater attention to health information. The mean age (67.4 ± 6.9 years) and proportion living alone (14%) were also lower than in earlier studies (9), which reported 70.93 ± 5.51 years and a 22% rate of solitary living. These findings align with previous research (9), which also identified age, residence, and cohabitation as key influencing factors.

In terms of digital health literacy dimensions, mean item scores from highest to lowest were: information access and evaluation (3.43 ± 1.03), application (2.00 ± 1.09), and interaction (1.49 ± 0.71). Yang et al. (39) found a similar pattern: access and evaluation (2.43 ± 1.23), application (2.28 ± 1.17), interaction (2.05 ± 1.21). Liu (9), however, reported higher scores for interaction than application: access and evaluation (2.89 ± 1.71), interaction (2.18 ± 1.56), and application (1.51 ± 1.03).

This inconsistency may reflect contextual factors. In our setting, the hospital actively promotes digital health services, including volunteer support and user training, helping patients use online tools for appointments and payments. Despite these efforts, participants still reported low willingness and ability to engage in digital interactions. Many people lacked confidence. In using digital tools, one fears operational mistakes and privacy breaches. Concerns over personal data exposure and potential harassment led some to avoid online engagement entirely. As a result, participants viewed themselves more as passive information recipients than active communicators, which may explain the higher application score relative to interaction.

#### 4.1.2 Digital health literacy among older adult patients with chronic diseases: three latent profiles identified

This study identified three distinct latent profiles of digital health literacy among older adult patients with chronic diseases: C1—Low Literacy–Passive Interaction, C2—Moderate Literacy–Limited Interaction, and C3—High Literacy–Active Interaction, reflecting marked heterogeneity in this population.

Category C1 (*n* = 142, 26.5%) demonstrated a low overall literacy level, with a mean score of 1.69 ± 0.3. Dimension scores were: 2.05 ± 0.5 (information access and evaluation), 1.24 ± 0.5 (application ability), and 1.04 ± 0.1 (interaction ability)—the lowest. Patients were mostly from rural or township areas, used the internet less than 1 h daily, perceived digital health information as “not at all/slightly useful,” and found it “very/somewhat difficult” or “neutral” to use. They also had higher adjusted Charlson Comorbidity Index (aCCI) scores. These individuals typically lacked digital skills, used smartphones mainly for entertainment or communication, and passively consumed health content. Influenced by frequent reports of online fraud, they lacked objective assessments of digital safety, often believing that browsing posed little risk while deeper engagement might lead to data breaches or financial loss. As a result, they preferred receiving rather than sharing information—aligning with prior research (9). Social alienation is common in this group and may contribute to reduced willingness or capacity for online interaction (40).

Category C2 (*n* = 276, 51.5%) had a moderate literacy level, with a mean score of 2.83 ± 0.4. Dimension scores were: 3.69 ± 0.5 (information access and evaluation), 1.72 ± 0.6 (application ability), and 1.35 ± 0.4 (interaction ability)—again, the lowest. This profile was defined as Moderate Literacy–Limited Interaction. Most participants lived in townships, had attended chronic disease education sessions, and used the internet for fewer than 4 h daily. They rated digital health information as “slightly useful” and “somewhat difficult” or “neutral” to use. These patients had relatively high self-efficacy and aCCI scores, strong social support, and moderate awareness of digital health risks. Aging-related cognitive decline makes adaptation to digital tools challenging, but younger seniors are more receptive to learning. Strong support systems enable access to information and technical help, boosting self-efficacy and encouraging engagement, even when actual interaction ability remains limited.

Category C3 (*n* = 118, 22.0%) had the highest overall literacy, with a mean score of 3.89 ± 0.3. Dimension scores were: 4.5 ± 0.39 (information access and evaluation), 2.36 ± 0.8 (application ability), and 3.56 ± 0.8 (interaction ability). This profile was labeled High Literacy–Active Interaction. Patients in this group were more likely to have attended health education programs and reported high levels of self-efficacy and social support. These individuals often received professional guidance from healthcare providers, demonstrated confidence in accessing and using digital tools, and actively engaged in health-related communication. Category C2 (*n* = 276, 51.5%) had a moderate literacy level, with a mean score of 2.83 ± 0.4. Dimension scores were: 3.69 ± 0.5 (information access and evaluation), 1.72 ± 0.6 (application ability), and 1.35 ± 0.4 (interaction ability)—again, the lowest. This profile was defined as Moderate Literacy–Limited Interaction. Most participants lived in townships, had attended chronic disease education sessions, and used the internet for fewer than 4 h daily. They rated digital health information as “slightly useful” and “somewhat difficult” or “neutral” to use. These patients had relatively high self-efficacy and aCCI scores, strong social support, and moderate awareness of digital health risks. Aging-related cognitive decline makes adaptation to digital tools challenging, but younger seniors are more receptive to learning. Strong support systems enable access to information and technical help, boosting self-efficacy and encouraging engagement, even when actual interaction ability remains limited. Category C3 (*n* = 118, 22.0%) had the highest overall literacy, with a mean score of 3.89 ± 0.3. Dimension scores were: 4.5 ± 0.39 (information access and evaluation), 2.36 ± 0.8 (application ability), and 3.56 ± 0.8 (interaction ability). This profile was labeled High Literacy–Active Interaction. Patients in this group were more likely to have attended health education programs and reported high levels of self-efficacy and social support. These individuals often received professional guidance from healthcare providers, demonstrated confidence in accessing and using digital tools, and actively engaged in health-related communication.

### 4.2 Influencing factors of digital health literacy profiles

This study found that residence, participation in chronic disease education, internet usage, perceived usefulness and ease of digital health tools, self-efficacy, and social support significantly influenced digital health literacy profiles in older adult patients.

#### 4.2.1 Individual level factors

Patients in C1 often perceived digital health information as difficult to use and of limited value. Prior studies show that perceived usefulness and ease of use strongly influence technology adoption (9). Most older adult individuals possess only basic digital skills and remain affected by the digital divide, with limited access to or ability to apply health technologies (41). Lower education, reduced literacy, minimal exposure to digital media, and limited device proficiency contribute to poor digital health literacy and complicate chronic disease self-management (42). Younger older adult patients are generally more adaptable, and as digital health technologies proliferate, new forms of ageism may emerge (43). Digital health literacy declines with age and is negatively associated with trust in digital sources and perceived usability (9, 44, 45). Frequent use of multiple digital devices is linked to quicker adoption and improved literacy (46). Significant differences in disease perception were observed among the three profiles (*p* < 0.05). C1 patients often lacked accurate awareness of disease progression, held negative perceptions, and were more prone to anxiety or distress (47), reducing their motivation to engage with digital resources (48). Conversely, C3 patients demonstrated higher self-efficacy, enabling them to apply health information effectively in chronic disease management, consistent with previous findings (11). Prior successful experiences may have built their confidence in using digital tools. They were also more inclined to share personal experiences, fostering improved digital literacy. However, some older adults remain reluctant to share health information due to concerns about accuracy, privacy, or social stigma, which may hinder engagement.

#### 4.2.2 Interpersonal relationships

Family support is the most immediate source of emotional and informational assistance for older adult patients. Younger family members generally possess higher levels of digital literacy. In families with strong intergenerational relationships, higher education levels, and financial stability, digital engagement and efficacy tend to be greater. Older adult individuals in such environments receive timely emotional and informational support when encountering difficulties accessing digital health resources. This support enhances their acceptance of digital technologies, improves self-efficacy, and reduces anxiety related to technology use (38, 49). However, in multigenerational households, older adult patients often assume caregiving roles or handle household responsibilities. These obligations consume time and energy, reducing their capacity to learn digital skills and improve digital health literacy. Peer support also plays a key role. Older adult patients tend to trust members of their social circles or fellow patients, which facilitates communication and mutual understanding. Information shared within these networks is perceived as vetted and reliable. Such peer interactions serve as both guidance and motivation, encouraging older adult individuals to develop their digital health literacy.

#### 4.2.3 Social support systems

Social support—emotional, informational, and instrumental—reduces stress and promotes overall well-being among older adult patients. It significantly influences their willingness to adopt digital technologies and their level of digital health literacy, aligning with previous findings (42, 50, 51). Most older adult individuals seek care at nearby community health centers or pharmacies, where long-term relationships with primary healthcare providers offer trusted emotional and informational support. Patients who have participated in chronic disease health education are more likely to fall into the high-literacy group (C3). These individuals tend to receive professional guidance from healthcare providers, demonstrate higher health literacy, and better understand their own information needs. They are more proactive in seeking health information, communicating, and engaging with digital health platforms (52). China’s digital health sector is expanding rapidly, supported by improved infrastructure and technologies. Digital media now plays a major role in disseminating health information (8), creating a favorable environment for older adult access to digital health resources (53). However, the field still lacks standardized regulation. Some search engines and short video platforms prioritize paid content through ranking algorithms, resulting in uneven information quality. Additionally, frequent device updates, complex interfaces, and online scams raise concerns about data privacy and financial safety. These factors undermine trust and hinder older adult patients’ digital health engagement.

## 5 Limitations

This study has several limitations that may affect both internal and external validity. First, the sample was drawn exclusively from older adult patients attending tertiary hospitals in Anhui Province, which may limit the generalizability of the findings to individuals in rural areas or those receiving care in primary or secondary healthcare settings. Tertiary hospitals generally provide better access to digital infrastructure and health services, which may positively skew digital health literacy levels. Second, all participants were required to possess basic proficiency in using smartphones or other smart devices. This inclusion criterion may have inadvertently excluded individuals most affected by the digital divide—those with minimal or no experience using digital technologies—thus potentially overestimating the actual level of digital health literacy in the broader older adult population. Third, the study employed a cross-sectional and once-off survey design, which limits the ability to draw causal inferences from the observed associations. Longitudinal studies are warranted to verify the directionality and potential causality of the relationships among digital health literacy, self-efficacy, and related influencing factors. Fourth, all data were collected using self-administered questionnaires based on self-perceptions, which may be subject to common method bias, including social desirability and recall bias. Finally, although the digital health literacy scale and other instruments used in this study have been validated in Chinese populations, they are not widely adopted in international research. This may limit the comparability of findings across different cultural or healthcare contexts. Future studies should consider incorporating internationally recognized tools to enhance cross-cultural applicability and validity.

## 6 Conclusion

This study found that older adult chronic disease patients generally have low levels of digital health literacy. Using latent profile analysis (LPA), patients were classified into three categories: C1: “Low Literacy - Passive Interaction Type,” C2: “Medium Literacy - Limited Interaction Type,” and C3: “High Literacy - Active Interaction Type,” revealing significant heterogeneity among the groups. Healthcare providers should prioritize patients in category C1, and design targeted intervention plans based on the characteristics of each group to enhance digital health literacy. In the context of digitalization and technological advancements, improving the digital health literacy of older adult chronic disease patients is a complex, multi-faceted task that requires the collective effort of society.

## Data Availability

The datasets presented in this article are not readily available because the dataset generated and analyzed during the current study contains personal health information from older adult patients with chronic diseases, and its use is subject to strict confidentiality agreements. Due to privacy and ethical concerns, the data cannot be made publicly available. Access to the dataset is restricted and available only upon reasonable request, with approval from the ethics committee of the participating institutions, in compliance with applicable data protection regulations. Requests to access the datasets should be directed to Xiumu Yang, 0700013@bbmu.edu.cn.
